# Galectin-1 is essential for efficient liver regeneration following hepatectomy

**DOI:** 10.18632/oncotarget.9194

**Published:** 2016-05-05

**Authors:** Tamara Potikha, Ezra Ella, Juan P. Cerliani, Lina Mizrahi, Orit Pappo, Gabriel A. Rabinovich, Eithan Galun, Daniel S. Goldenberg

**Affiliations:** ^1^ The Goldyne Savad Institute of Gene Therapy, Hadassah-Hebrew University Medical Center, Jerusalem, Israel; ^2^ Department of Pathology, Hadassah-Hebrew University Medical Center, Jerusalem, Israel; ^3^ Laboratory of Immunopathology, Institute of Biology and Experimental Medicine, CONICET, Buenos Aires, Argentina; ^4^ Faculty of Exact and Natural Sciences, University of Buenos Aires, Buenos Aires, Argentina

**Keywords:** galectin-1, liver regeneration, hepatectomy, lipid metabolism, Pathology Section

## Abstract

Galectin-1 (Gal1) is a known immune/inflammatory regulator which acts both extracellularly and intracellularly, modulating innate and adaptive immune responses. Here, we explored the role of Gal1 in liver regeneration using 70% partial hepatectomy (PHx) of C57BL/6 wild type and Gal1-knockout (Gal1-KO, *Lgals1−/−*) mice. Gene or protein expression, in liver samples collected at time intervals from 2 to 168 hours post-operation, was tested by either RT-PCR or by immunoblotting and immunohistochemistry, respectively. We demonstrated that Gal1 transcript and protein expression was induced in the liver tissue of wild type mice upon PHx. Liver regeneration following PHx was significantly delayed in the Gal1-KO compared to the control liver. This delay was accompanied by a decreased Akt phosphorylation, and accumulation of the hepatocyte nuclear p21 protein in the Gal1-KO versus control livers at 24 and 48 hours following PHx. Transcripts of several known regulators of inflammation, cell cycle and cell signaling, including some known PHx-induced genes, were aberrantly expressed (mainly down-regulated) in Gal1-KO compared to control livers at 2, 6 and 24 hours post-PHx. Transient steatosis, which is imperative for liver regeneration following PHx, was significantly delayed and decreased in the Gal1-KO compared to the control liver and was accompanied by a significantly decreased expression in the mutant liver of several genes encoding lipid metabolism regulators. Our results demonstrate that Gal1 protein is essential for efficient liver regeneration following PHx through the regulation of liver inflammation, hepatic cell proliferation, and the control of lipid storage in the regenerating liver.

## INTRODUCTION

Galectin-1 (Gal1) is a β-galactoside-binding lectin, encoded by the *Lgals1* gene, which is widely expressed in multiple cell types including immune cells and acts both extracellularly and intracellularly, modulating innate and adaptive immune responses. This lectin blunts inflammatory responses by promoting apoptosis of activated, but not resting T cells, suppressing the secretion of pro-inflammatory cytokines and favoring secretion of anti-inflammatory IL-10 [1, 2]. Gal1 is also a key effector of regulatory T cells and suppresses chronic inflammation in different experimental models [1, 3]. Over-expression of Gal1 in different tumor types and/or their associated stroma promotes tumor progression through multiple mechanisms including inhibition of efficient anti-tumor immune response [1, 4], augmentation of Ras activation [5], stimulation of tumor angiogenesis [6, 7], and activation of p38 MAPK, ERK1/2 and COX-2 signaling pathways [8]. Interestingly, nuclear factor (NF)-κB controls expression of Gal1, which may in turn attenuate activation of this transcription factor through a self-regulatory mechanism [9]. Moreover, recent studies identified a role for Gal1 as a compensatory mechanism that preserves angiogenesis in anti-VEGF refractory tumors by co-opting the VEGFR2 signaling pathway [10], suggesting that it may mimic canonical ligands to sustain signaling pathways in different biological processes.

Expression of Gal1 is associated with the aggressiveness of hepatocellular carcinoma (HCC) in mice [11], low survival of HCC patients [12], and poor prognosis in HCC following resection [13]. Interestingly, Gal1 acts by promoting HCC cell adhesion through PI3K and/or ERK1/2 signaling pathways [14]. In murine HCC models, we have demonstrated that an inefficient anti-inflammatory activity of the endogenous Gal1 is associated with increased inflammation at an early age and with enhanced tumor development at an older age [15]. On the other hand, the tumor-promoting effect of the hepatitis C virus (HCV) transgene in the chronic inflammation-mediated HCC mouse model, was associated with increased Gal1 expression in the liver [16]. Notably, this lectin can also either activate or inhibit cell proliferation depending on cell type and cell activation status [1]. Based on the multiple activities of Gal1, we investigated whether Gal1 controls hepatocarcinogenesis, at least in part, through direct effects on hepatocyte proliferation in the injured liver.

Here we have identified a role for Gal1 in liver regeneration (LR) following partial hepatectomy (PHx). LR following 70% PHx is a highly ordered and well studied process of compensatory hyperplasia which restores the liver mass by a combination of hepatocyte proliferation and hypetrophy [17]. This process is controlled by three main partially overlapping and redundant networks: cytokines, growth factors, and metabolic regulators [18, 19]. There is a constantly growing list of so called auxiliary mitogens which typically are not mitogenic for hepatocytes *in vivo* or *in vitro* when supplemented directly, although their absence causes a significant delay in LR [20]. We found that Gal1 deficiency results in a significant retardation of LR following PHx, through mechanisms involving selective regulation of PHx-induced genes including inflammatory mediators as well as regulators of cell cycle and lipid metabolism.

## RESULTS

### Delayed recovery of liver mass in Gal1-KO mice following partial hepatectomy

**Figure 1:**
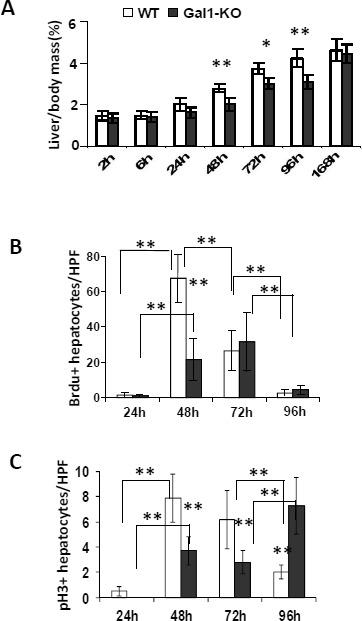
Delayed liver regeneration following PHx in Gal1-KO (*Lgals1^−/−^*) mice **A.** Liver-to-body weight ratios (%) in WT *versus* Gal1-KO mice at different time points following PHx. **B.** Quantification of the BrdU-positive hepatocyte nuclei at 24 to 96 hours following PHx. **C.** Quantification of hepatocyte nuclei positive for phosphorylated histone 3 (pH3) in regenerating livers post-PHx. **B.** and **C.** on “y” axis - average numbers of positive nuclei per one HPF; at least 10 HPFs were counted per each liver sample. Standard deviation and statistical significance (*t*-test) are shown: *, *P* < 0.05 and **, *P* < 0.005; 5 - 6 males per each experimental group.

To explore the potential role of Gal1 in the regulation of hepatocyte proliferation, we used PHx as a well-established method for studying LR. Isogenic wild type (WT) and Gal1-KO mice underwent either 70% PHx or sham surgery and were sacrificed at 2, 6, 24, 48, 72, 96 and 168 hours after operation. Liver and serum samples were collected for subsequent analysis. Restoration of the liver mass following PHx was significantly attenuated in Gal1-KO mice as compared to WT mice at 48 to 96 hours post-operation (Figure 1A). Nevertheless, at 168 hours (7 days) following PHx, the liver mass in the Gal1-KO mutants was completely restored, as in the WT mice (Figure 1A). Notably, body weights of both groups of mice recovering from PHx were comparable (data not shown). Monitoring markers of the proliferative machinery demonstrated a reduced level of BrdU incorporation into hepatocyte nuclei of Gal1-KO compared to WT mice at 48 hours following PHx, while the situation reversed at 96 hours following PHx (Figure 1B, Supplementary Figure 1A). In parallel, phosphorylation of histone H3, which is a highly specific marker of mitosis, was significantly reduced in the hepatocyte nuclei of Gal1-KO compared to WT mice at 48 and 72 hours following PHx, while it was significantly increased in mutants at 96 hours following PHx (Figure 1C, Supplementary Figure 1B). Thus, loss of Gal1 resulted in a significant retardation of hepatocyte DNA synthesis, of hepatocyte proliferation, and of restoration of liver mass following PHx.

### Absence of Gal1 results in aberrant expression of cell cycle regulatory proteins in the regenerating mutant liver

**Figure 2:**
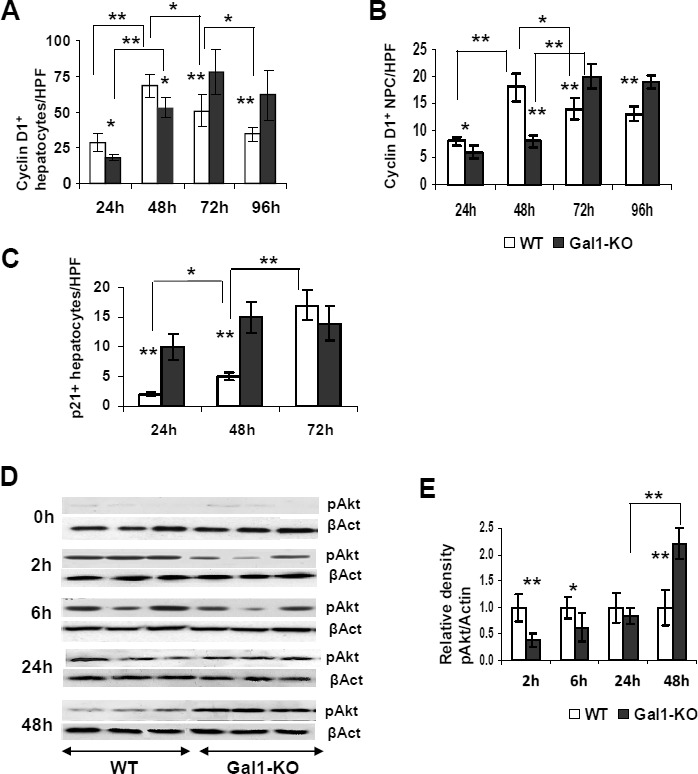
Aberrant expression of several cell cycle related proteins in the liver of WT and Gal1-KO mice following PHx **A.**, **B.** Quantification of cyclin D1-positive nuclei in hepatocytes **A.** and non-parenchymal cells **B.** of the Gal1-KO *versus* WT post-pHx livers, assessed by IHC. **C.** Quantification of the p21-positive hepatocyte nuclei at 24, 48 and 72 hours following PHx. **A.**, **B.**, **C.** on “y” axis - average numbers of positive nuclei per one HPF; at least 10 HPFs were counted per each liver sample; 6 males per each experimental group. **D.** Phosphorylation of the Akt protein was evaluated by immunoblotting of the whole liver homogenates obtained from WT and Gal1-KO mice at 2, 6, 24 and 48 hours post-PHx (upper panel for each time point); β-actin (βAct) was used as a loading control (bottom panel for each time point). **E.** Densitometric analysis of the pAkt protein expression presented at **D.** each pAkt band was normalized to the matched β-actin band followed by normalization of each Gal1-KO value to the matched WT value. Three to four males per each experimental group; standard deviation and statistical significance (*t*-test) are shown: *, *P* < 0.05; **, *P* < 0.005.

To explore the possible molecular mechanisms underlying the delayed DNA replication and hepatocyte proliferation in the regenerating livers of Gal1-KO mice, we compared the expression of the cyclin D1, p21 and phosphorylated Akt proteins in WT and Gal1-KO regenerating livers (Figure 2 & Supplementary Figure 2). Concomitantly with the reduction of hepatocyte proliferation in Gal1-KO mice *versus* WT mice, a significant decrease in the levels of cyclin D1 was observed in both hepatocytes and non-parenchymal cells of the mutant liver at 24 and 48 hours post-PHx. However, at 72 and 96 hours post-PHx, cyclin D1 expression was significantly higher in all cell types of Gal1-KO compared to WT mice (Figure 2A, 2B; Supplementary Figure 2A). Level of the p21 protein, which is believed to inhibit cell cycle progression, was significantly increased in hepatocyte nuclei of Gal1-KO *versus* WT mice at 24 and 48 hours post-PHx (Figure 2C; Supplementary Figure 2B). As expected, p21 expression was not detected in the naïve and sham-operated livers of both congenic strains (data not shown).

We also tested the phosphorylation status of several important mitogenic signaling proteins, essential for cell proliferation. We found no difference in the phosphorylation of STAT3, mitogen activated protein kinases (MAPKs) and p38 between Gal1-KO and WT livers at the time points studied following PHx (data not shown). Meanwhile, there was a noticeable variation in the phosphorylation (and activation) of the Akt protein in Gal1-KO relative to the WT liver within 2 and 48 hours post-operation: Akt phosphorylation was significantly reduced at 2 and 6 hours, while it was significantly increased at 48 hours post-PHx in the mutant liver (Figure 2D, 2E). The aberrant expression patterns of cyclin D1, p21 and phosphorylated Akt in the Gal1-KO livers following PHx are in accordance with the observed retardation of liver mass recovery and hepatocyte proliferation in mutant mice, thus underscoring the essential role of Gal1 in the regulation of critical intracellular signals and molecular markers associated with hepatocyte proliferation during LR.

### Gal1 induction following PHx

**Figure 3:**
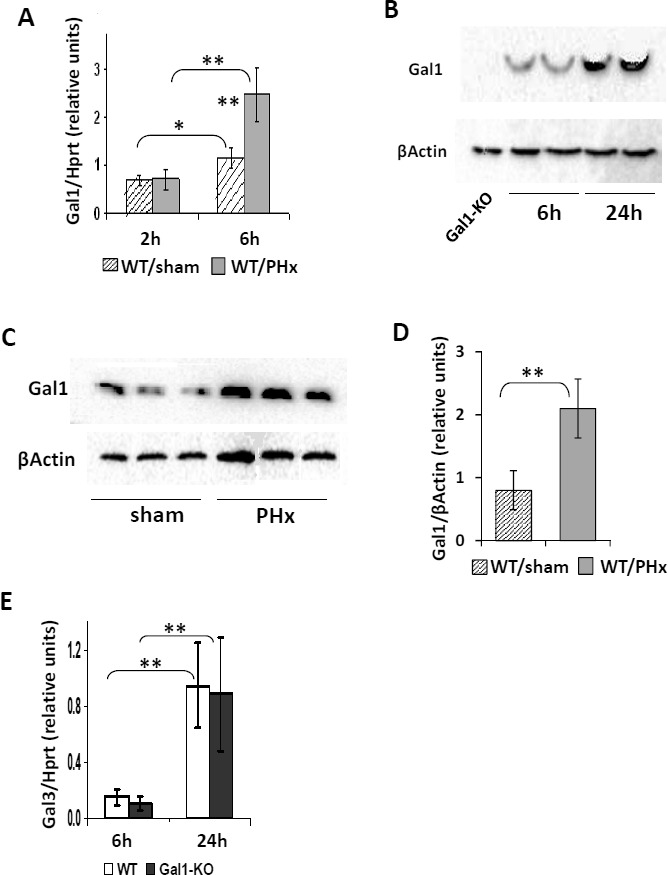
Induction of the Gal1 gene (*Lgals1*) in regenerating WT livers early following PHx **A.** Quantification of the Gal1 transcript level in WT livers at 6 hours post-PHx and in to sham-operated livers by qRT-PCR. **B.** and **C.** Testing the Gal1 protein level (~14.5 kDa) in WT livers at 24 hours following PHx compared to either 6 hours following PHx **B.** or sham-operated controls at 24 hours following PHx **C.** by immunoblotting. **D.** Quantification of data shown in **C.**. E. Lack of Gal1 does not affect the expression of the galectin-3 (Gal3, *Lgals3*) gene at either 6 or 24 hours following PHx (qRT-PCR). In all experiments: 3 - 4 male mice per experimental group; statistical significance (*t*-test): *, *P* < 0.05, **, *P* < 0.005.

We examined whether Gal1 is induced in WT mice during the first 24 hours of LR following 70% PHx, and found a significant induction of the Gal1 transcript in the regenerating post-PHx compared to the sham-operated liver at 6, but not at 2 hours following operation (Figure 3A). The level of Gal1 protein in the liver of hepatectomized mice was not increased at 6 hours following PHx (not shown), but it was significantly increased at 24 hours compared to both hepatectomized mice at 6 hours post-PHx (Figure 3B) and to sham-operated controls at 24 hours post-PHx (Figure 3C, 3D, Supplementary Figure 3A). Increased Gal1 protein expression was detected also by IHC in the post-PHx compared to the sham-operated liver of WT mice at all tested time points following operation (Supplementary Figure 3B). In both sham-operated and post-PHx WT livers, Gal1 was expressed mainly by endothelial cells, by lymphocytes, and by pericentral hepatocytes. Given the established role of galectin-3 in regulating cell proliferation [21], we examined whether altered galectin-3 expression could compensate for the loss of the Gal1 protein in LR. Hepatic expression of galectin-3 was similarly increased in Gal1-KO and WT livers both at 6 and at 24 hours post-PHx (Figure 3E). These findings demonstrate that Gal1 is induced during the first day of LR following PHx in WT mice, and that its loss is not compensated by altered galectin-3 expression.

### Decreased inflammation in the livers of Gal1-KO mice one day following PHx

**Figure 4:**
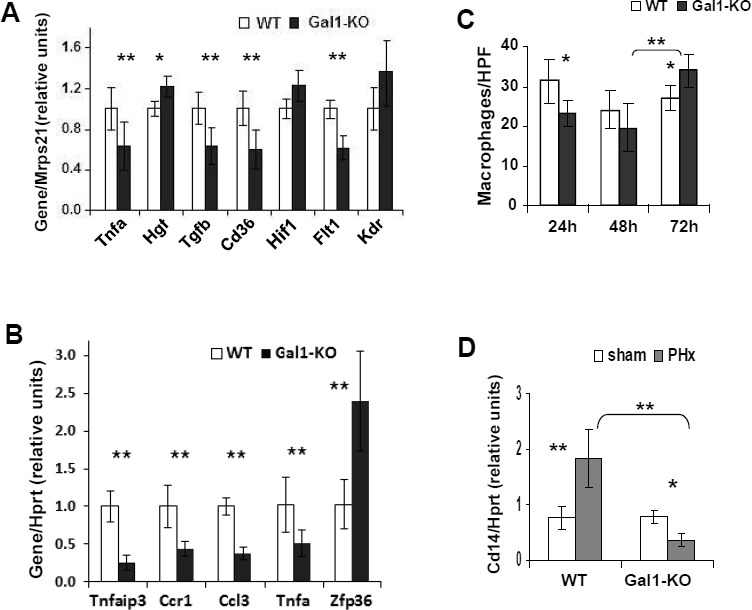
Decreased hepatic inflammation in Gal1-KO mice at the early stages of LR following PHx **A.**, **B.** Graphical representations of differential expression of the tested inflammation- and angiogenesis-related genes in the regenerating WT and Gal1-KO livers at 24 hours post-PHx. A. Semi-qRT-PCR, in triplicates. B. qRT-PCR analysis of liver gene expression, in triplicates (RQ - relative quantification). **C.** Histomorphometric quantification of monocytes/macrophages in the liver is represented as an average number of F4/80-positive non-parenchymal cells per one HPF. **D.** Expression of the *Cd14* transcript in Gal1-KO and WT livers at 24 hours following either PHx or sham surgery (qRT-PCR). In all experiments: 3 - 4 male mice per experimental group; reference genes: *Mrps21* - in **A.**, *Hprt* - in **B.** and in **D.**; statistical significance (*t*-test): *, *P* < 0.05; **, *P* < 0.005.

Examination of the liver enzyme activity in the serum of WT and Gal1-KO mice during LR revealed no significant differences between the genotypes except at 72 hours following PHx, when the levels of ALP decreased in Gal1-KO *versus* WT mice, possibly indicating a reduced biliary damage in the mutant mouse liver (Supplementary Figure 4A, 4B). Sham-operated mice did not exhibit any signs of hepatocyte or biliary damage (shown only at 2 and 6 hours post-PHx). The frequency of apoptotic cells during LR (measured by TUNEL assay) was also similar between the WT and Gal1-KO livers, with very few apoptotic hepatocytes detected in both groups (data not shown). Thus, the overall level of liver tissue injury following PHx was not affected by Gal1 loss. Based on the established anti-inflammatory activity of Gal1, we evaluated the expression of several inflammation-related genes in the regenerating WT and Gal1-KO livers at 24 hours post-PHx using both semi-qRT-PCR (Figure 4A, Supplementary Figure 5A) and qRT-PCR (Figure 4B, Supplementary Figure 5B). Unexpectedly, the genes *Tnfa*, *Cd36, Ccr1, Tnfaip3,* and *Tgfb* were found to be down-regulated, while *Zfp36* was up-regulated in Gal1-KO compared to WT livers (Figure 4A, 4B; genes that were not affected by Gal1 loss are shown in Supplementary Figure 5B). Interestingly, *Flt1* (*Vegfr1*, encoding receptor for vascular endothelial growth factor 1) was also significantly down-regulated in the Gal1-KO liver. As a control, we also tested Gal1 expression in these experimental groups, at 24 hours post-PHx: both semi-qRT-PCR and qRT-PCR detected the Gal1 transcript only in the WT, but not in the Gal1-KO liver (not shown).

**Figure 5:**
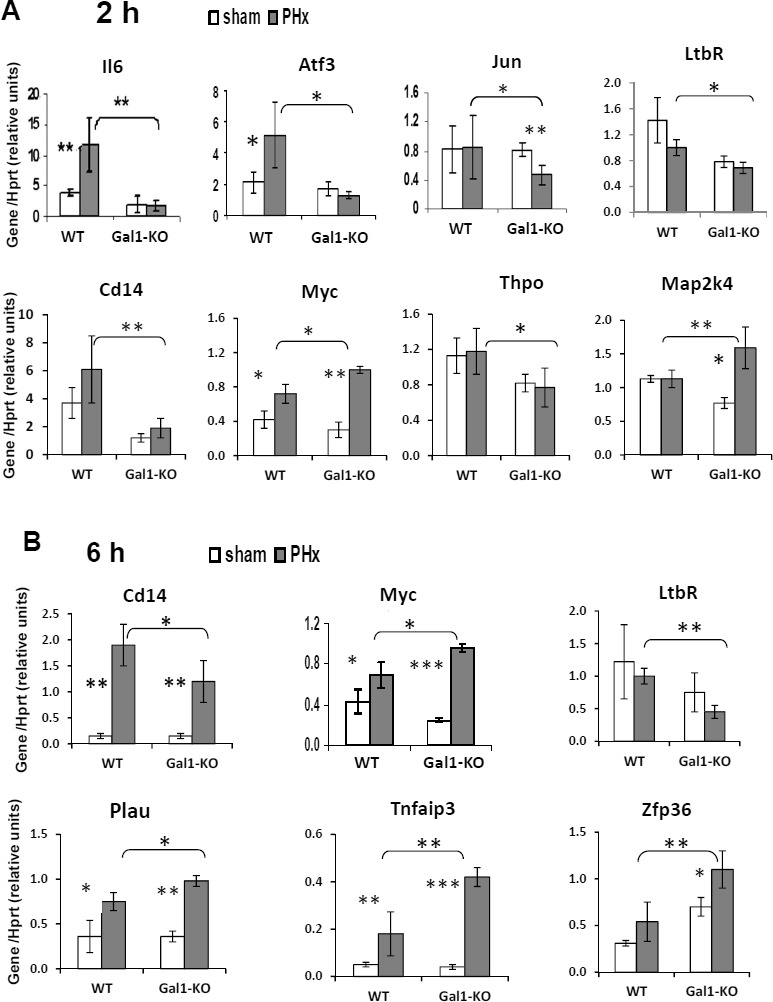
Aberrant expression of genes, known to be induced in LR, in the liver of Gal1-KO compared to WT mice Gene expression in the liver at 2 hours **A.** and 6 hours **B.** following either PHx (grey color) or sham (white color) surgeries. Real-Time RT-PCR using *Hprt* gene expression as an internal control; 4 - 5 male mice per experimental group. In all experiments, data represent mean ± SD; statistical significance (*t*-test): *, *P* < 0.05, **, *P* < 0.02, ***, *P* < 0.005.

Given the importance of monocytes and macrophages in LR, we then assessed their infiltration into the liver parenchyma by immunostaining for F4/80, a marker of mature mouse macrophages and blood monocytes. The number of macrophages and monocytes was decreased in the Gal1-KO *versus* WT livers at 24 hours, while it was substantially increased at 72 hours following PHx (Figure 4C and Supplementary Figure 6). Monocytes and macrophages accumulated mainly in the portal tracts of both congenic livers following PHx. Remarkably, the expression of the *Cd14* gene, which is expressed mainly by macrophages and, to a lesser extent, by neutrophils, was significantly reduced in Gal1-KO compared to WT livers at 2, 6 and 24 hours post-PHx (Figure 5A, 5B and Figure 4D). The expression of the *Ccl3* (*Mip1α*) gene, which encodes a chemokine promoting macrophage chemoattraction and influencing wound healing and inflammation, was significantly down-regulated in the Gal1-KO compared to the WT liver at 24 hours post-PHx (Figure 4B). Thus, Gal-1 may control LR, at least in part, through regulation of inflammatory responses.

### Gal1 deficiency influences the expression of multiple genes in the early stages of LR following PHx

We then studied whether Gal1 deficiency alters the expression of genes known to be induced at the very early stages of LR following PHx using qRT-PCR. For this purpose, we selected 32 genes that are involved in the control of inflammation and cell proliferation (many of them are known as PHx-induced genes; Table 1). Lack of Gal1 affected the expression of eight genes at 2 hours post-PHx (Figure 5A) and of six genes at 6 hours post-PHx (Figure 5B); only *Cd14, Ltbr* and *Myc* were affected at both time points. Two hours post-PHx, six genes were found to be down-regulated and two were found to be up-regulated in Gal1-KO compared to WT liver (Figure 5A), whereas at 6 hours post-PHx, only two genes were down-regulated while four were up-regulated in the Gal1-KO compared to the WT liver (Figure 5B). However, only in six tested cases, the difference in gene expression between mutants and controls reached or exceeded the two-fold threshold: Gal1 absence caused a three-fold down-regulation of the genes *Atf3, Cd14* and *Il6* at 2 hours post-PHx, and a 2.5-fold down-regulation of the gene *Ltbr* with a two-fold up-regulation of the genes *Zfp36* and *Tnfaip3* at 6 hours post-PHx. Four among these six genes have well established roles in the regulation of LR. IL-6 is one of the main pro-inflammatory and regeneration-promoting cytokines in the priming phase of LR, which induces the expression of about 40% of the immediate early genes in the regenerating liver [18]. Atf3 (Lrf-1, liver regenerating factor 1) induces DNA synthesis and cyclin D1 expression [22]. Both *Il6* and *Atf3* genes were induced already at 2 hours post-PHx in the WT, but not in the Gal1-KO liver (Figure 5A). Signaling through Ltbr (receptor for lymphotoxin- β) is essential for efficient LR, specifically - for initiation of DNA synthesis [23]. Tnfaip3 (A20) is a potent inhibitor of inflammation and of NF-κB activation [24].

**Table 1:** RT-PCR analysis of liver gene expression following PHx in the Gal1-KO compared to WT mice

Genes			Hours post-PHx			Il6-induced	Inflam-mation	Lipid meta-bolism	Other
Gene symbol	Gene ID NCBI	Alternative name	2	6	24	2 h ^#^			
Acly	104112	Atpcl	−	down	NC			V	
Atf3	11910	Lrf1	down	NC	NC				V
Ccl3	20302	Mip1a	−	NC	down		V		
Ccna1	12427	Cyclin A	−	−	NC				V
Ccr1	12768	Rantes-R	−	−	down		V		
Cd14	12475		down	down	down	V	V		
Cd36	12491	Fat	−	−	NC*		V	V	
Cidec	14311	Fsp27	−	NC	down			V	
Crem	12916	Icer	NC	NC	−				V
Egr1	13653	N_gfi_-A	NC	NC	NC	V	V		
Fabp4	11770	Afabp	−	down	down			V	
Flt1	14254	Vegfr1	−	−	down*				V
Fos	14281	c-Fos	NC	NC	−	V			V
Gadd45a	13197	Ddit1	NC	NC	NC	V			V
Hgf	15234	Sf	−	−	NC*				V
Hif1a	15251	Mop1	−	−	NC*				V
Igfbp1	16006	Ibp1	NC	NC	−	V			V
Il6	16193	Ifnb2	down	NC	NC		V		
Jun	16476	Ap-1c	down	NC	NC	V			V
Kdr	16542	Vegfr2, Flk1	−	−	NC*				V
Lgals1	16852	Galectin-1	ND	ND	ND*		V		
Lgals3	16854	Galectin-3	−	NC	NC		V		
Lta	16992	Lymphotoxin α	ND	ND	-		V		
Ltb	16994	Lymphotoxin β	NC	NC	NC		V		
Ltbr	17000	Lymphotoxin β receptor	down	down	NC		V	V	
Map2k4	26398	Jnkk1, Mek4	Up	NC	NC	V			V
Myc	17869	c-Myc	Up	Up	NC	V			V
Plau	18792	u-PA	NC	Up	−	V			V
Plin4	57435	S3-12	−	down	down			V	
Ppara	19013	Ppar, Nr1c1	NC	NC	NC			V	
Saa1	20208	Saa-1	−	NC	NC		V		
Socs3	12702	Cis3	−	NC	NC		V		
Tgfb1	21803	Tgf-beta1	NC	NC	down*				V
Thpo	21832	Thrombopoietin	down	NC	−	V			V
Tnf	21926	Tnf-alpha	NC	NC	down		V		
Tnfaip3	21929	A20	NC	Up	down	V	V		
Tnfsf14	50930	Light, Hveml	NC	NC	NC		V		
Zfp36	22695	Tristetraprolin	NC	Up	Up	V	V		

NC - not changed (comparable expression in WT and Gal1-KO post-PHx livers)

ND - not detected; Up – up-regulated; down – down-regulated; − not tested

* tested by semi-qRT-PCR; others were tested by qRT-PCR “Up” or “down” at least two-fold in Gal1-KO compared to WT post-PHx liver is shown in bold

# genes whose induction at 2 h post-PHx was IL6-dependent [Li, 2001 #1370].

### Loss of Gal1 results in aberrant lipid metabolism in the regenerating mutant liver

**Figure 6:**
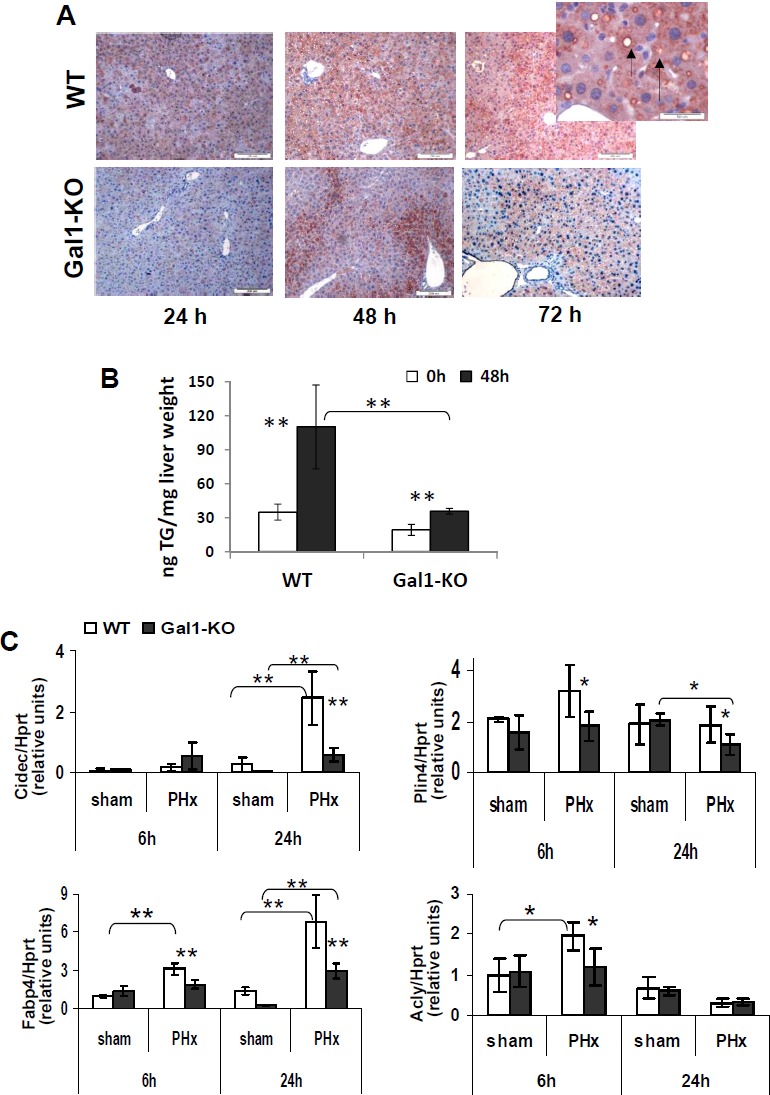
Significantly delayed and decreased transient steatosis in the Gal1-KO compared to WT liver following PHx **A.** Immunohistochemical staining of liver sections for adipophilin (arrow) in the Gal1-KO compared to WT liver at 24, 48 and 72 hours following PHx. Magnification x100; insert x400. **B.** Levels of triglycerides in the Gal1-KO compared to WT liver samples at 48 hours following PHx (Triglyceride Quantification Colorimetric/Fluorometric Kit #K622, Biovision Inc., Milpitas, CA; three males per each experimental group); **C.** Expression of adipogenic genes in the Gal1-KO compared to WT liver at 6 and/or 24 hours post-PHx. Real time RT-PCR using *Hprt* gene expression as the internal control; 4 - 5 male mice per group. Data represent mean ±SD; statistical significance (*t*-test): *, *P* < 0.05, **, *P* < 0.02.

Comparative histological analysis of regenerating livers revealed a significantly reduced temporal steatosis in the Gal1-KO compared to control WT mice at 24, 48, and 72 hours following PHx (Supplementary Figure 7A). Immunohistochemical staining of liver sections for adipophilin (perilipin 2, a specific marker of lipidogenesis encoded by the gene *Plin2*) revealed a reduced expression of this protein showing a one-day delay in its appearance in the Gal1-KO compared to WT post-PHx livers (Figure 6A). There was a negligible staining for adipophilin in the naïve livers of both congenic strains (data not shown) as well as in the sham operated livers of both congenic strains (Supplementary Figure 7B). Direct quantification of the triglycerides level in liver tissue samples demonstrated a significantly reduced level of triglycerides in Gal1-KO compared to WT liver at 48 hours post-PHx (Figure 6B).

To explore the underlying molecular mechanisms of aberrant lipidogenesis in Gal1-KO livers, we examined the expression of *Cidec*, *Fabp4*, *Plin4*, and *Acly* genes which are responsible of regulating this process, in the liver of both strains (Figure 6C). While the Cidec (Fsp27) protein promotes the formation and enlargement of lipid droplets [25], Fabp4, the fatty acid binding protein, functions in the fatty acid uptake, transport, and metabolism, and the Plin4 protein, similar to Plin2, coats intracellular lipid storage droplets. On the other hand, Acly (ATP citrate-lyase) is a cytosolic enzyme responsible for the synthesis of acetyl-CoA in many tissues and connects glucose/glutamine metabolism to the *de novo* lipid synthesis [26]. The expression of all four tested genes was significantly reduced in the Gal1-KO compared to the control WT liver: *Acly* - at 6 hours, *Cidec* - at 24 hours, while *Fabp4* and *Plin4* - both at 6 and 24 hours post-PHx (Figure 6C). The most significant alteration (at least, two-fold) was a decrease of *Cidec* and *Fabp4* expression at 24 hours post-PHx. These two genes were induced in both genotypes at 6 and 24 hours post-PHx, compared to sham-operated mice. Interestingly, aberrant lipidogenesis in the Gal1-KO post-PHx liver correlated with the reduced size of hepatocytes in mutants compared to controls at 48 and 72 hours following operation, as revealed by immunohistochemical staining of liver sections for β-catenin (Supplementary Figure 8).

## DISCUSSION

The endogenous lectin Gal1 is mainly known for its regulatory role in the regulation of immune cell programs, inflammatory responses and angiogenesis; however it has also been implicated in the control of cell survival, signaling and proliferation, acting in different model systems either as a mitogen or as an inhibitor of cell proliferation [1, 11]. Here we identified a novel role for Gal1 in LR following PHx. Our findings reveal that Gal1 is induced already at 6 hours post-PHx, and is essential for an efficient LR by stimulating different processes in the liver, including early inflammation, hepatocyte proliferation, liver adipogenesis and angiogenesis (Supplementary Figure 9). Interestingly, previous studies showed that Gal1 promotes peripheral nerve regeneration by different molecular mechanisms, including macrophage stimulation [27, 28]. In line with these findings, we demonstrate here that Gal1 deficiency significantly reduced the recruitment of monocytes/macrophages during the first 24 hours post-PHx (Figure 4C, 4D) and resulted in a decreased expression of the genes *Tnfa,* encoding TNFα, one of the main regulators of LR, and *Ccl3*, encoding a chemokine Mip1α that promotes monocyte/macrophage chemoattraction (Figure 4A, 4B). The earliest effects of Gal1 loss in the regenerating liver were the absence of induction of the *Atf3*, *Il6* and *Cd14* genes at 2 hours post-PHx (Figure 5A). Moreover, at 6 hours post-PHx, loss of Gal1 caused a decreased expression of *Ltbr* and an increased induction of the genes *Tnfaip3* and *Zfp36* both of which encode potent inhibitors of inflammation (Figure 5B). Remarkably, during the first 24 hours post-PHx, loss of Gal1 resulted mainly in a reduced induction or expression of multiple genes; only two genes, *Tnfaip3* and *Zfp36,* both negative regulators of inflammation, were up-regulated in the Gal1-KO hepatectomized liver (Table 1).

Interestingly, *Tnfaip3* encodes the ubiquitin-modifying enzyme A20 which restricts the duration and intensity of NF-κB signaling and, in turn, is induced by NF-κB [24]. This negative feedback of NF-κB signaling is common for both A20 and Gal1 [9]. Recently, it was demonstrated that A20 promotes LR by decreasing SOCS3 expression and enhancing the IL-6/STAT3 proliferation signaling pathway [29]. Thus, down-regulation of the *Tnfaip3* expression at 24 hours post-PHx (Figure 4B) could be one of the reasons for a retarded LR in the Gal1-KO liver. We detected no changes in the expression of the *Socs3* and *Saa1* genes in the Gal1-KO liver at 24 hours post-PHx (Table 1 and Supplementary Figure 5B; *Saa1* has been tested as an established target of the IL-6/STAT3 signaling). Nevertheless, p21 protein, whose expression is known to be down-regulated in response to an excess of A20 [30], was found to be up-regulated in the post-PHx Gal1-KO liver (Figure 2C), in agreement with the reduced *Tnfaip3* expression at 24 hours post-PHx. Thus, our data demonstrate that absence of Gal1 causes an aberrant response of the *Tnfaip3* gene encoding A20 protein on PHx: up-regulation at 6 and down-regulation at 24 hours post-operation, suggesting a decreased NF-κB signaling which promotes LR at this stage.

*Zfp36* encodes tristetraprolin, which negatively regulates the expression of multiple genes, including many cytokines, mainly by promoting a rapid decay of their transcripts [31]. Among its known targets are *Tnfa*, *Ccl3* and *Csf2*, encoding the granulocyte-macrophage colony-stimulating factor GM-CSF [31]. Thus, a pronounced up-regulation of *Zfp36* in Gal1-KO compared to the WT liver both at 6 and 24 hours post-PHx (Figure 5B, Figure 4B), could be one of the factors responsible for the down-regulation of *Tnfa* and *Ccl3* and for the delayed monocyte/macrophage recruitment in mutants at 24 hours post-PHx (Figure 4). Despite the well-known up-regulation of *Zfp36* expression early following PHx [32], its function in LR has not been explored. We suggest that tristetraprolin, A20 and Gal1, represent a group of stress-induced regulators of inflammation and cell proliferation with partially overlapping functions. The well-known redundancy of LR mechanisms may explain the early induction of *Zfp36* and *Tnfaip3* in the absence of Gal1.

IL-6, a well-established pro-inflammatory and pro-angiogenic cytokine, has been identified as a key feature of LR [18]. Notably, the induction of *Il6* expression was abolished in the Gal1-KO liver at 2 hours post-PHx, but was comparable to that in the WT liver at 6 and 24 hours post-PHx (Figure 5A, Table 1). Because some studies confirm the importance of IL-6 for LR following PHx, while others do not - the precise roles of IL-6 in LR still remain uncertain [18]. Here we have examined, among others, the expression of 11 genes that were previously shown to be induced at 2 hours post-PHx in an IL-6-dependent fashion [32]. Despite the lack of *Il6* induction in the Gal1-KO liver at 2 hours post-PHx, the IL-6-dependent genes did not show a defined pattern of expression at either 2 or 6 hours post-PHx: some of them were up-regulated, some - down-regulated, and some had similar expression levels in mutants and controls (Table 1). Thus, it is difficult to delineate the precise consequences of the absence of early *Il6* induction in the Gal1-KO post-PHx liver. If IL-6 regulates some genes in concert with Gal1 (or a Gal1-dependent regulator), then, in the absence of Gal1, such genes may turn to be IL-6-independent. In this regard, recent studies revealed that the microbe-driven IL-6 inflammation contributes to tumor progression through Gal1-dependent mechanisms [33].

The most significant effects of Gal1 loss on the cell cycle machinery were the reduced Akt phosphorylation at 2 and 6 hours post-PHx (Figure 2D, 2E), the absence of *Atf3* induction at 2 hours post-PHx (Figure 5A), and the decreased cyclin D1 and increased p21 levels at 24 and 48 hours post-PHx (Figure 2A-2C). Akt is phosphorylated (and activated) early in LR following PHx by TNFα and by growth factor signaling [18]. Whereas Akt is activated by IL-6 [34] the lack of IL-6 induction at 2 hours post-PHx could be one of the factors contributing to the diminished Akt activation in the Gal1-KO liver. Although in non-hepatocyte cells, p21 phosphorylation by Akt prevents its association with PCNA, which inhibits DNA replication [35], in LR, activated Akt stimulates the restoration of hepatocyte size and may promote hepatocyte proliferation only in the absence of p21 [36]. The *Atf3* gene encodes the activating transcription factor 3 which is also called LR factor 1 (LRF-1). The absence of its induction in the Gal1-KO liver early post-PHx might contribute to a retarded DNA synthesis and to decreased levels of the cyclin D1 protein, since Atf3 directly activates its transcription in hepatocytes [22]. The significantly increased expression of nuclear p21 in the hepatocytes of Gal1-KO mice at 24 and 48 hours post-PHx might be one of the main factors responsible for the retardation of LR in mutants.

Our findings showing a decreased accumulation of lipids in the Gal1-KO liver post-PHx demonstrate for the first time the influence of an endogenous lectin on lipid metabolism in the liver. Transient hepatic steatosis in the regenerating liver is a well-known phenomenon; despite highly controversial studies on the significance of specific lipid metabolism genes for an efficient LR, most published data show that altered lipid metabolism, including accumulation of lipid droplets, in the post-PHx liver is functionally important for the initiation of LR [37]. At the molecular level, we demonstrated that Gal1 loss resulted in a decreased transcript expression for several genes encoding proteins that are associated with the formation of lipid droplets. Interestingly, we found a reduced expression of adipophilin (perilipin-2), in the liver of Gal1-KO mice during first three days following PHx (Figure 6A). The functional relevance of this protein in LR following PHx has been confirmed recently by the demonstration of delayed LR in Plin2-KO mice [38]. Hence, the insufficient lipid accumulation in the post-PHx mutant liver could be an additional factor responsible for the retardation of LR in Gal1-KO mice. Remarkably, we found a significantly reduced expression of the *Ltbr* gene (but not of its ligands’ genes) in the early post-PHx stage (Figure 5, Table 1). The *Ltbr* gene encodes the lymphotoxin- β receptor which controls both LR (specifically - initiation of DNA synthesis) and lipid metabolism in the liver [23, 39].

It is well known that the functional activity of galectins significantly depends on their carbohydrate ligands [40]. Although the impact of Gal1 carbohydrate ligands has been explored in several cells and tissues, their relevance in liver physiology is less understood. In this regard, recent studies have shown that complex branched N-glycans, structures that are generated by the Mgat5 glycosyltransferase and serve as Gal1 ligands, play key roles in glycemic responses to exogenous glucagon in hepatocytes. In fact, glucagon receptor signaling and gluconeogenesis were impaired in Mgat5−/− cultured hepatocytes [41]. Moreover, analysis of hepatocarcinoma cells revealed substantial glycan changes during epithelial mesenchymal transition, including increased β1-6 N-glycan branching and decreased α2,6 sialylation, suggesting higher exposure of Gal1-specific glycoepitopes during this protumorigenic process [42]. Finally, Mgat5-mediated β-1-6-GlcNAc branched N-glycosylation conferred hepatic cells with resistance to anoikis through EGFR/PAK1 activation [43].

Finally, we found that Gal1 loss resulted in the reduced expression of the *Flt1* (*Vegfr1*) transcript encoding VEGFR1 (Figure 4A, Supplementary Figure 5A). In this regard, the pro-angiogenic activity of Gal1 has been identified mainly in the context of tumor neo-vascularization under hypoxic conditions [6, 7], and contributed to VEGFR2, but not to VEGFR1 signaling due to the differential sialylation of these glycosylated receptors [10]. In conclusion, our results demonstrate that Gal1 expression is induced early following 70% PHx, and that Gal1 is essential for an efficient LR through the activation of multiple molecular pathways. Gal1 loss resulted in a significantly delayed hepatocyte proliferation and liver mass restoration, which were accompanied by a delay in monocyte/macrophage recruitment, and a decrease in transient liver post-PHx steatosis (Supplementary Figure 9). Based on the well known redundancy of signaling networks directing LR, we propose that Gal1 could act hierarchically in this process in concert with other regulators of inflammation, cell cycle, lipid metabolism and angiogenesis. Further analysis is required to elucidate the complex interactions of these signaling networks in LR, and the functional relevance of the Gal1-glycan axis in physiologic and pathologic liver conditions.

## MATERIALS AND METHODS

Full details are available in the Supplementary Methods.

### Mice

Mice were maintained at the Specific Pathogen-Free unit of the Animal Facility, The Hebrew University Medical School, under a 12 h light/dark cycle, and provided with food and water *ad libitum*. All animals received human care, and all animal study protocols were approved by the Hebrew University-Hadassah Medical School Ethics Review Board (the Animal Care Unit holds National Institutes of Health (NIH) approval number OPRR-A01-5011 and the American Association for the Accreditation of Laboratory Animal Care International accreditation number 1285). Wild type C57Bl/6 (B6) mice were obtained from Harlan farm (Hebrew University, Israel); the Lgals1−/− (Gal1-KO) mutants of the B6 strain were kindly provided by Prof. Francoise Poirier (Institut Jacques Monod, Universités P6 and P7, Paris, France). The 70% PHx or sham surgery was performed as described by Greene and Puder [44].

### Isolation and analysis of total liver RNA and total liver protein

Total RNA was isolated from frozen liver tissues using Trizol reagent (Invitrogen, Carlsbad, CA) as described by the manufacturer. Gene expression was evaluated either by semi-qRT-PCR [15], or by real-time RT-PCR as described in the Supplementary Methods using primers described in Supplementary Table 1. Total liver protein was isolated and analyzed by immunoblotting as previously described [15].

### Immunohistochemistry and counting of positive cells

Immunostaining was done on 4-μm-thick formalin-fixed paraffin-embedded liver tissue sections by standard procedures. Antibodies used in this study and their antigen retrieval procedures are shown in Supplementary Table 2. Cas-Block™ (008120, Invitrogen, Carlsbad, CA, USA) was used for dilution of all antibodies as well as for tissue blocking. The following HRP-conjugated secondary antibodies were used: anti-rabbit (K4003), anti-mouse (K4001; both Envision, Dako, Denmark), anti-rat (Histofine, Nishirei, Japan). Color was developed using either 3-amino-9-ethylcarbazole (AEC, 00-1111, Invitrogen) for 10 min (30 min in the case of CD3) (Gal-1, CD3, Ly6B, BrdU) or 3,3′-diaminobenzidene using a Zymed Super Picture kit (87-9663, Invitrogen, CA, USA) for 5 min (β-catenin, cyclin D1, F4/80, p21). Counter stain was performed with filtered Cat-Haematoxylin (Pharmatrade, UAE). Negative controls were used by omitting the primary antibody, or using a Gal-1-KO liver in the case of Gal1. The stainings were visualized with the Nikon Eclipse E600 microscope equipped with the CellSens Entry imaging software (Olympus, Australia). Histological assessment of liver slides was performed by a clinical pathologist (O.P.) in a blind fashion. The number of F4/80+, cyclin D1, p21-expressing cells for each slide was calculated as the sum in the x20 or x40 microscope power field. Ten fields per slide were included, and a total of 4 or 5 mice per group were used.

### Immunoblotting

Samples from the whole liver tissue lysates (50 μg protein/lane) were resolved on SDS-PAGE (Hoefer Mighty small SE245, Pharmacia Biotech, USA) and electrophoretically transferred (Trans-Blot Semi-dry, BioRad) onto PVDF membranes (Bio-Rad, CA, USA). Gal1 protein was detected with the above mentioned rabbit anti-Gal1 (1:5000). Mouse β-actin (1:200, Abcam, UK) was used as a control to monitor sample loading. The immunocomplexes were visualized using the anti-rabbit (1:300), or anti-mouse (1:200) antibodies described above for 45 min and the EZ-ECL detection system (Biological Industries, Beit Ha-Emek, Israel) or Amersham ECL Prime (RPN2232, GE Healthcare UK Ltd, England) for 5 min.

### Quantification of triglycerides in liver samples

Frozen liver tissues (~100 mg) were homogenized in 1 ml solution containing 5% NP-40 in water, slowly heated to 80-100°C in a water bath for 2-5 min until the NP-40 became cloudy, then were cooled down to room temperature. The heating was repeated one more time to sollubilize all triglycerides. The samples were centrifuged for 2 min to remove any insoluble material and diluted 10-fold with dH_2_O before the assay, which was performed using the Triglyceride Quantification Colorimetric/Fluorometric Kit (# K622, Biovision Inc., Milpitas, CA). In the assay, TGs were converted to free fatty acids and glycerol. The glycerol was then oxidized to generate a product which reacts with the probe to generate color (spectrophotometry at λ = 570 nm).

### Statistical analysis

Statistical significance between groups was estimated using the two-tailed unpaired *t*test. Results are expressed as the mean ±standard error of the mean (SEM); differences were considered significant at *P* < 0.05.

## SUPPLEMENTARY MATERIALS FIGURES AND TABLES



## Abbreviations

PDK1: Akt, phosphoinositide-dependent kinase 1
ALT: alanine aminotransferase
ALP: alkaline phosphatase
Atf3: activating transcription factor 3
BrdU: bromodeoxyuridine
Gal-1: galectin-1
IL: interleukin
H&E: hematoxylin and eosin
HPF: high power field
KO: knockout
Lgals1: galectin-1 gene symbol
LR: liver regeneration
Ltbr: lymphotoxin beta receptor
NF-κB: nuclear factor kappa B
PHx: partial hepatectomy
Plin: perilipin
RT-PCR: reverse transcription polymerase chain reaction
qRT-PCR: quantitative (real-time) RT-PCR
Vegfr: vascular endothelial growth factor receptor
WT: wild type
