# Supplementary material for: Galectin-1 is essential for efficient liver regeneration following hepatectomy
Source: Oncotarget. 2016 May 5;7(22):31738–54. doi: 10.18632/oncotarget.9194 (PMC5077973; doi:10.18632/oncotarget.9194)
Supplement: Supplementary file 1 [file oncotarget-07-31738-s001.pdf]

Galectin-1 is essential for efficient liver regeneration following hepatectomy

Supplementary Material

Supplementary Table 1: List of primers used in this study.

Gene symbol	Alternative name	Sense primer	Antisense primer
Acly	Atpcl	GTCCCAAGTCCAAGATCCCTG	CGTCTCGGGAACACACGTAG
Atf3	Lrf1	TTACCGTCAACAACAGACCCC	GACACTTGGCAGCAGCAATTT
Ccl3	Mip1a	TACAGCCGGAAGATTCCACG	GTCAGGAAAATGACACCTGGC
Ccna1		AGTTCAGCTTGTAAGATATCCAGA	AAGGAAGAAGAAGAGAACTGCT
Cd14		CATTTGCATCCTCCTGGTTTCTGA	CATTTGCATCCTCCTGGTTTCTGA
Cd36	Fat	TGTGTTTGGAGGCATTCTCA	GAGCCCACTTCGCATTACAT
Cidec	Fsp27	ACGCCATGAAGTCTCTCAGC	GATGCCTTTGCGAACCTTCC
Egr1	Ngfi-A	CAGCGCCTTCAATCCTCAAG	CAGCGCCTTCAATCCTCAAG
Fabp4	Afabp	TGGGATGGAAAGTCGACCAC	CTTCCTTTGGCTCATGCCCT
Flt1	Vegfr1	TGAGGAGCTTTCACCG	TGAGGAGCTTTCACCG
Fos	Ap-1	CGGGGACAGCCTTTCCTACTA	GACAGATCTGCGCAAAAGTCCT
Gadd45a	Ddit1	CTGCAGAGCAGAAGACCGAA	GGGTCTACGTTGAGCAGCTT
Hgf	Hpta	CCTGAAAAGACCACTTGC	CCTGAAAAGACCACTTGC
Hif1a	Hif1α	AGCCTCACCAGACAGAGC	AGCCTCACCAGACAGAGC
Hprt		GCGATGATGAACCAGGTTATGA	ATCTCGAGCAAGTCTTTCAGTCCT
Igfbp1	Ibp1	TAGCTGCAGCCCAACAGAAA	CCAGGGATGTCTCACACTGTTT
Jun	cJun	TGAGTGCTAGCGGAGTCTTAACC	CCCCGCTTCTGTAACAAAGTTT
Kdr	Flk1, Vegfr2	TATCCCATTGGAGGAACC	TATCCCATTGGAGGAACC
Lgals1	Galectin-1	AATCATGGCCTGTGGTCTGG	TGGGCATTGAAGCGAGGATT
Lgals3	Galectin-3	ACTAATCAGGTGAGCGGCAC	AGGCATCGTTAAGCGAAAAGC

Lta	Lymphotoxin α	AGCAGCATCTTCTAAGCCCT	GTCATGTGGAGAACCTGCTGTG
Ltb	Lymphotoxin β	GGGACGTCGGGTTGAGAAG	ACGGTTTGCTGTCATCCAGT
Ltbr	Lymphotoxin β receptor	TCAAAGCCCAGCACAATGTC	TTATCGCATAGAAAACCAGACTTGC
Map2k4	Jnkk1	CCACAAACCAAGTGGGCAGA	ATGGGCAATCACTACTCCGC
Myc	cMyc	TTCCTTTGGGCGTTGGAAAC	GCTGTACGGAGTCGTAGTCG
Plau	Upar	CCTCTGGACCTGACTCCTGA	GAGGGCACACACATCCTCAA
Plin4	S3-12	GGGAAAAGGAAGAAGAAGAGAACTG	AGGCATCTTCACTGCTGGTC
Ppara	Ppar	CCTGAACATCGAGTGTCGAATAT	CCTGAACATCGAGTGTCGAATAT
Saa1	Tp53i4	GGTCTGGGCTTCTTCCTACC	CAGTTCTGAAACCCTTGTGGG
Socs3	Cis3	TAGACTTCACGGCTGCCAAC	CGGGGAGCTAGTCCCGAA
Thpo	Thrombopoietin	CCATGGCCCCAGGAAAGATT	TCAGTCAGCTCCATTCTGGC
Tnfa	Tnfα	CCACATCTCCCTCCAGAAAA	GTGGGTGAGGAGCACGTAGT
Tnfa	Tnfα	ATGGCCTCCCTCTCATCAGT	CTTGGTGGTTTGCTACGACG
Tnfaip3	A20	CAGTTTTGCCCACAGTTCCG	GCATGCATGAGGCAGTTTCC
Tnfsf14	Light, Hveml	CTGCCAGATGGAGGCAAAGG	CGTTGGCTCCTGTAAGATGTG
Zfp36	Tristetraprolin	GTGACAAGTGCCTACCTACCC	TCCCCACAGCAATGACGAGT

Supplementary Table 2: List of primary antibodies used in this study.

Epitope	Company	Catalogue number	Tissue retrieval and dilution	Secondary antibody
**Immunohistochemistry**				
Adipophilin (Adfp, Plin2)	LS Bio	3121	Citrate; 1/100	Anti-rabbit
Brdu	Dako	M 0744 (Bu20a)	Citrate; 1/50	Anti-mouse
Cd3	Serotec	MCA1477	EDTA; 1/100	Anti-rabbit
Cyclin D1	Diagnostic biosystems	RMA13003	glycine; 1/125	Anti-rabbit
F4/80	Abcam	Ab6640	pronase; 1/250	Anti-rat
Galectin-1 (Gal1, Lgals1)	produced by G.A.R.		Citrate; 1/500	Anti-rabbit
Ly-6B.2	Serotec	MCA771GA	Citrate; 1/3000	Anti-rat
p21	BD Pharmingen	556431	Citrate; 1/25	Anti-mouse
pH3	Millipore	06-570	Citrate; 1/250	Anti-rabbit
β-Catenin	BD Biosciences	610154	Citrate; 1/150	Anti-mouse
γH2Ax	Millipore	05-636	EDTA ; 1/30000	Anti-mouse
**Immunoblotting**				
pAkt (Ser473)	Cell Signalling	4060 (D9E)	Western; 1/2000	Anti-rabbit
p-p44/42 MAPK (Erk1/2) Thr202/Tyr204	Cell Signalling	4376 (20G11)	Western; 1/1000	Anti-rabbit
pStat3 (Tyr705)	Cell Signalling	9145 (D3A7)	Western; 1/1000	Anti-rabbit
Stat3(F2)	Santa Cruz	SC-8019	Western; 1/300	Anti-mouse
Beta-Actin	MP Biomedicals	691001	Western; 1/14000	Anti-mouse

Remarks: Citrate, pH 6.0; EDTA ,pH 8.0; glycine, pH 9.0; pronase = 1 mg/ml protease XIV (P8038, Sigma) for 10 min at R.T.

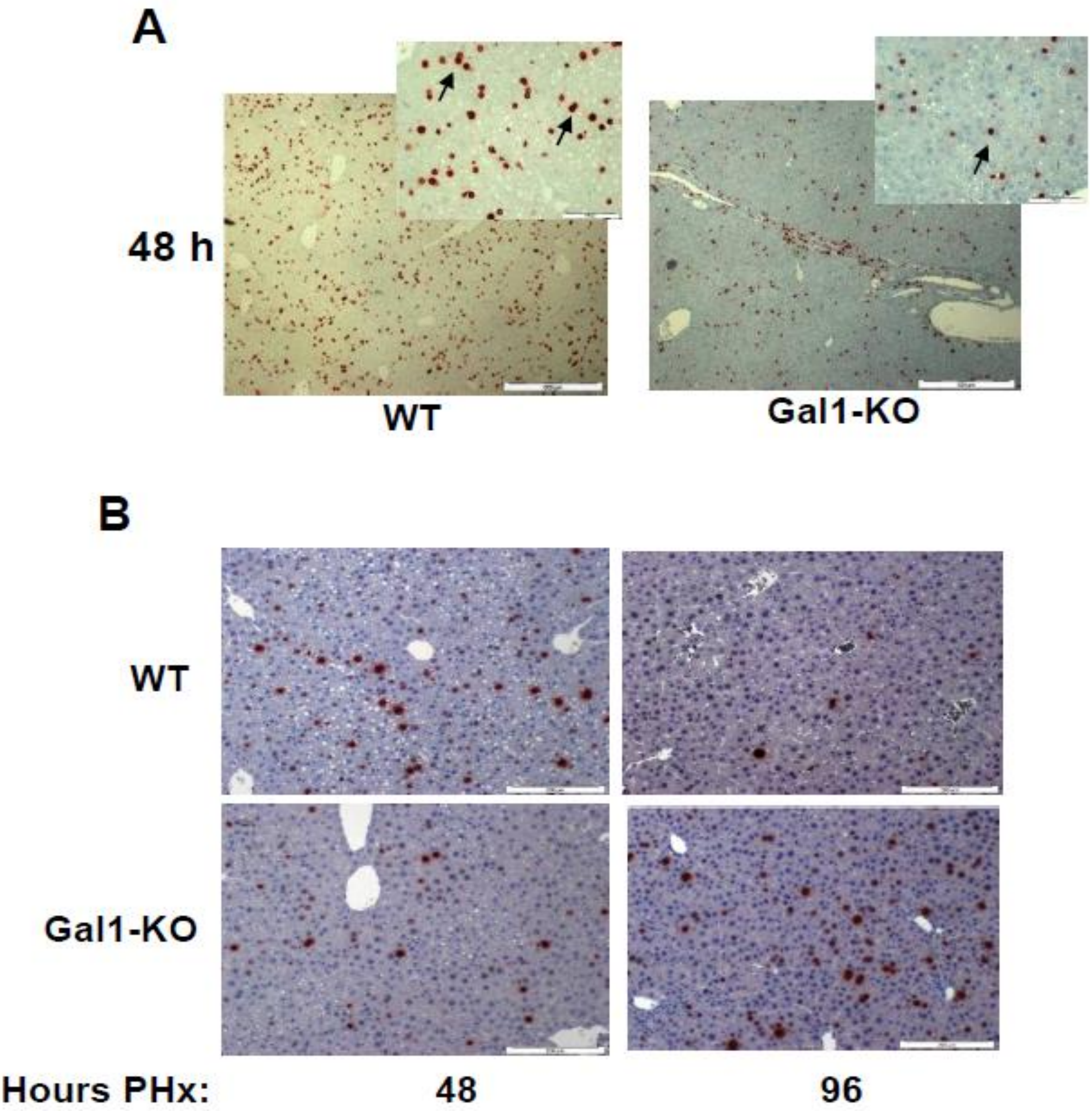

Supplementary Figure 1: **Markers of delayed liver regeneration following PHx in the Gal1-KO compared to control WT and mice. A.** Immunostaining of BrdU-positive cells in the regenerating liver. Representative sections from WT & Gal1-KO livers at 48 hours post-PHx are shown. Arrowheads indicate BrdU-positive hepatocyte nuclei (red color).

B. Immunohistochemical staining of the phosphorylated histone H3 as a marker of mitosis in regenerating livers of WT and Gal1-KO mice at 48 hours and 96 hours post-PHx; 5 – 6 males per each experimental group. Quantification of the results shown in (**A**) and (**B**) is presented in Figures 1B and 1C, respectively.

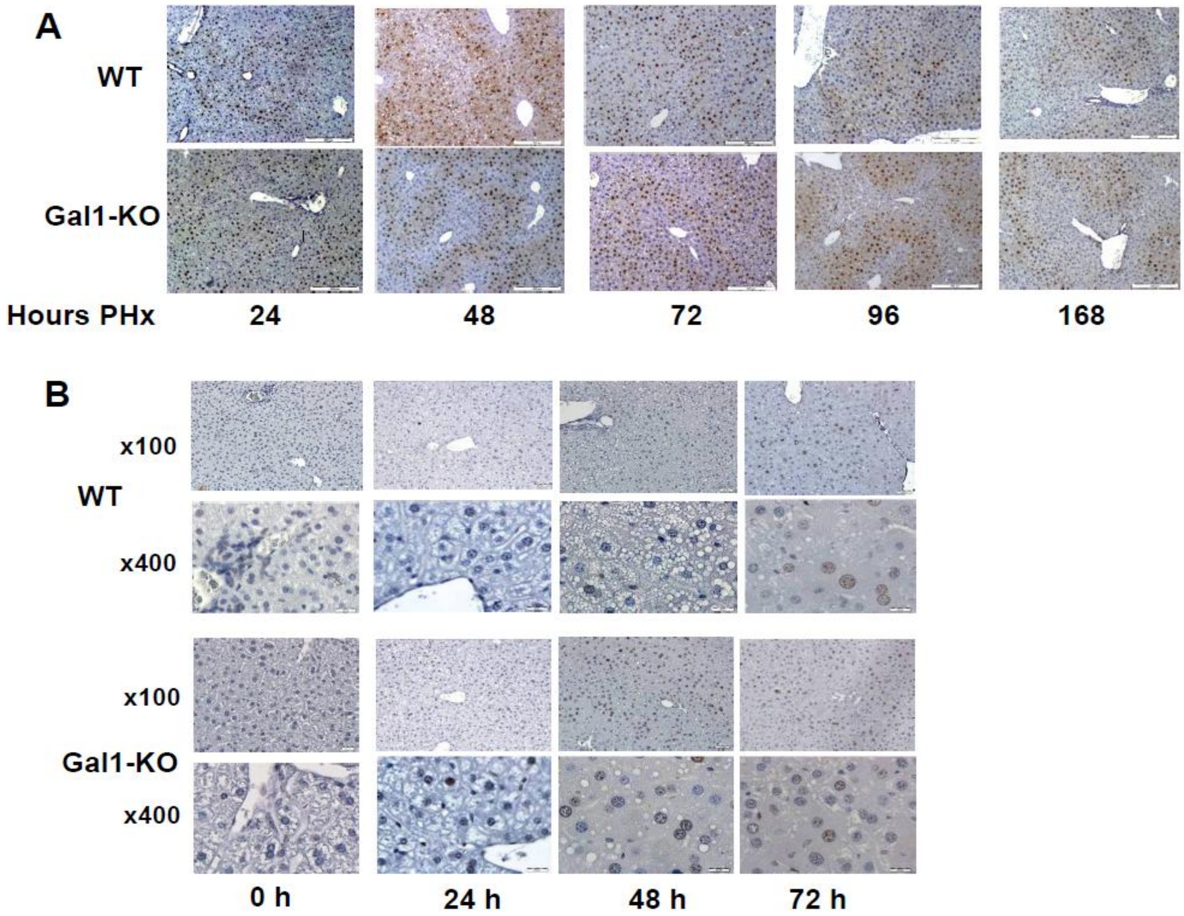

Supplementary Figure 2: Retarded LR of Gal1-KO compared to WT liver following PHx is associated with a decreased nuclear expression of cyclin D1 and an increased hepatocyte nuclear expression of p21 protein. A. Immunohistochemical staining of liver sections for cyclin D1 protein; magnification x100. **B.** Immunohistochemical staining of liver sections for p21 protein; magnification x400. There was no p21 nuclear immunostaining in the naive liver sections (time 0) of both congenic strains.

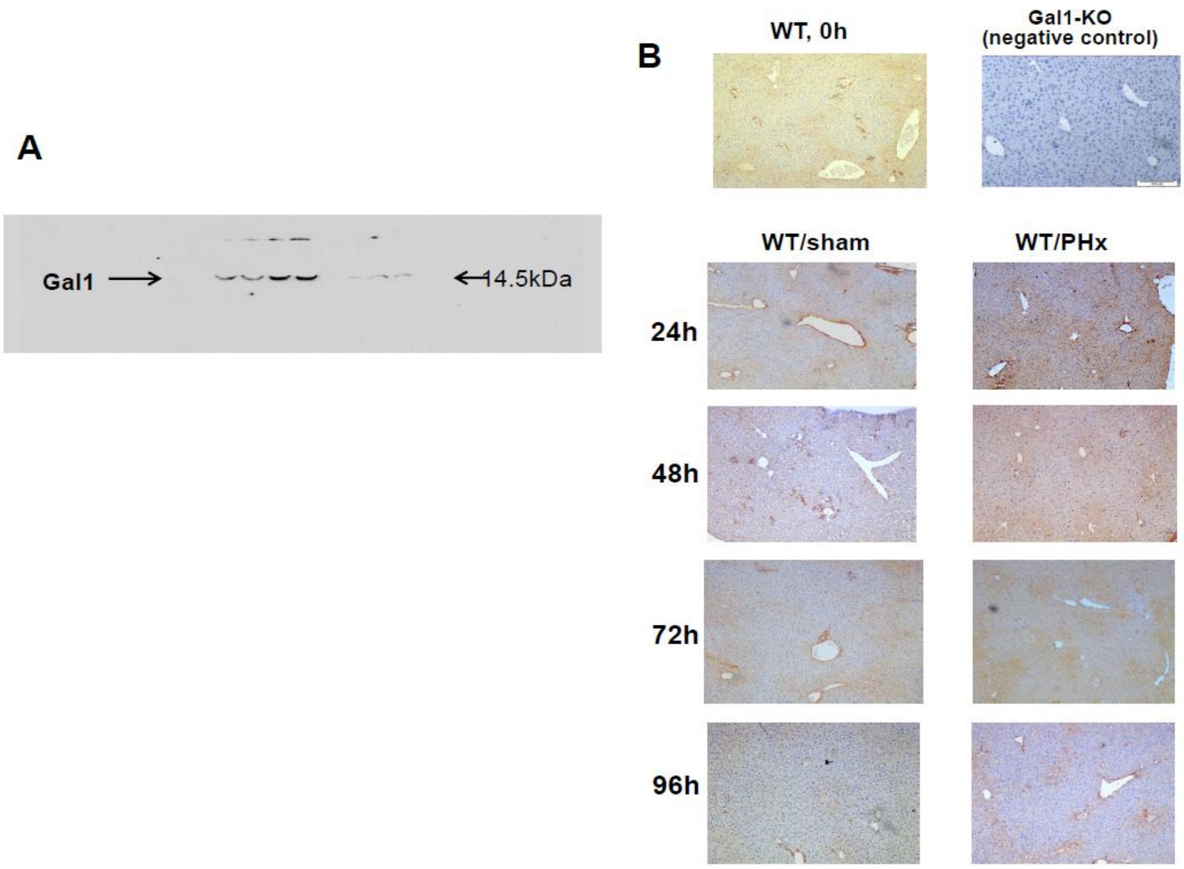

Supplementary Figure 3: Analysis of the Gal1 protein expression in the WT liver following PHx. A. Analysis of the Gal1 protein expression in WT livers at 24 hours compared to 6 hours following PHx by immunoblotting (the full size version of the blot presented in the Figure 3B). **B**. Immunohistochemical analysis of the Gal1 protein expression in WT livers at 1, 2, 3 and 4 days following PHx; 4 - 6 males per each time point and group (Gal1-KO liver serves as a negative control).

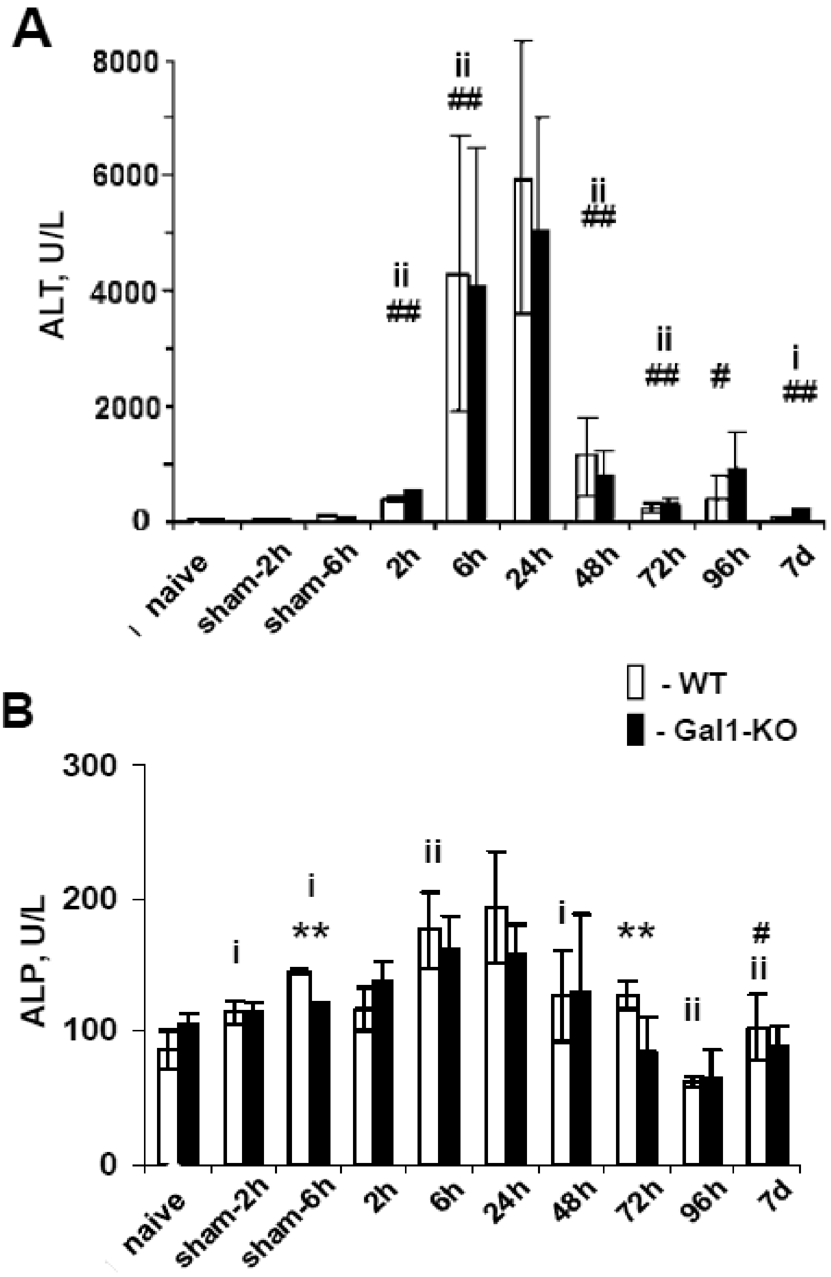

Supplementary Figure 4: **Markers of liver injury in serum of the control WT and Gal1-KO mice following PHx.** Activity of liver enzymes in the serum of WT and Gal1-KO mice at different time points following either PHx or sham surgery: ALT (**A**) and ALP (**B**). At least 6 males per each genotype and time point for naïve and PHx groups; 2 – 4 males per each sham-treated group. Standard deviation and statistical significance (*t*-test) are indicated: *, $P<0.05$ and **, $P<0.005$ in Gal1-KO vs. WT mice at indicated times post-PHx; i, $P<0.05$ and ii, $P<0.005$ for WT mice vs. a previous operation time point; $, $P<0.05$ and $$, $P<0.005$ for Gal1-KO mice vs. a previous operation time point.

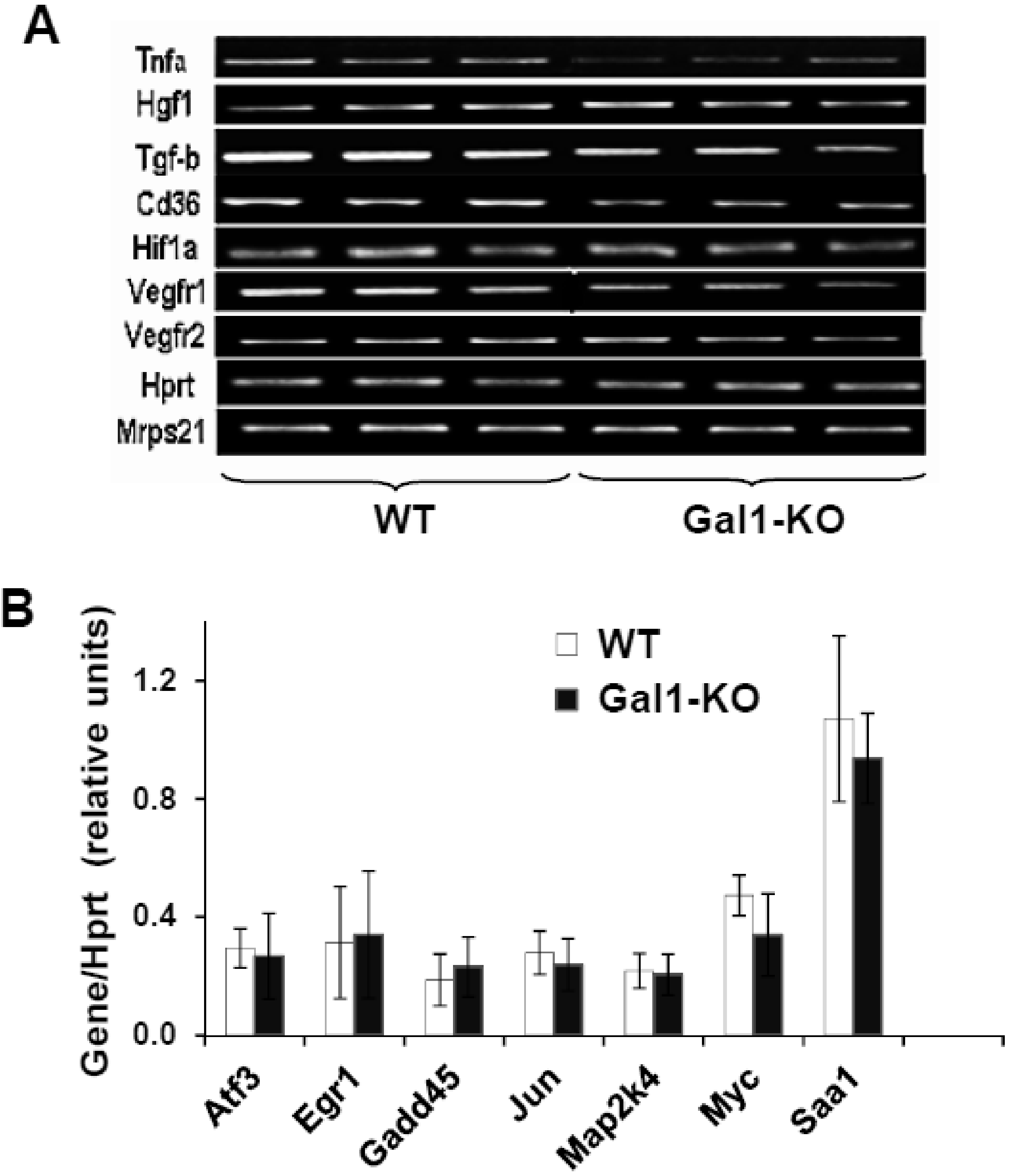

Supplementary Figure 5: Analysis of liver gene expression by RT-PCR. A. Differential expression of several inflammation- and angiogenesis-related genes in the regenerating WT and Gal1-KO livers at 24 hours post-PHx (semi-quantitative RT-PCR, in triplicates; *Mrps21* served as the reference gene). Quantification of these results is shown in Figure 4A. **B.** Real-time RT-PCR analysis of liver gene expression that was similar in the WT and Gal1-KO mice at 24 hours post-PHx.

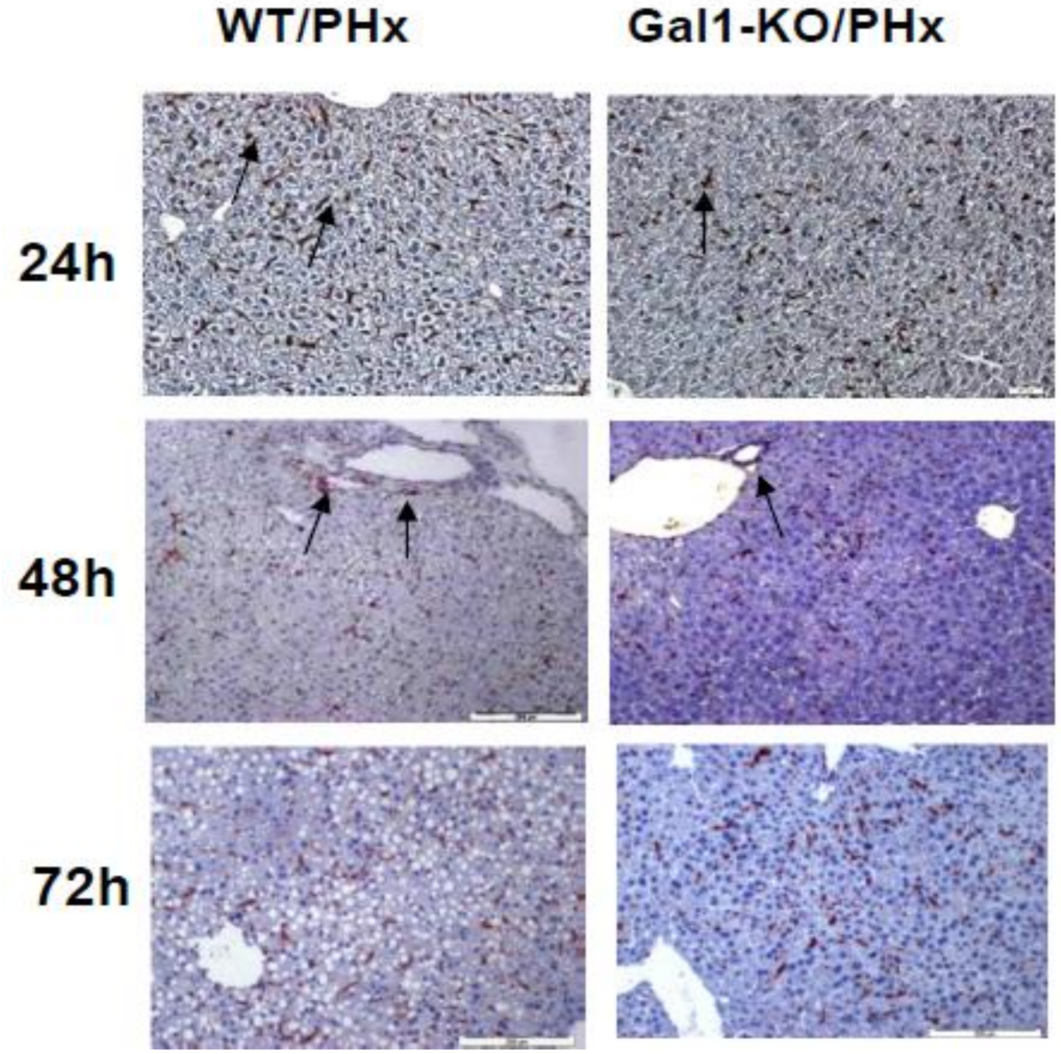

Supplementary Figure 6: Immunohistochemical analysis of monocyte/macrophage recruitment in the Gal1-KO compared to WT liver following PHx using the F4/80 marker. Macrophages/monocytes at indicated time points following PHx are shown by arrows; quantification of these results is shown in Fig. 4C. Magnification x100; 4 - 6 male mice per each time point and group.

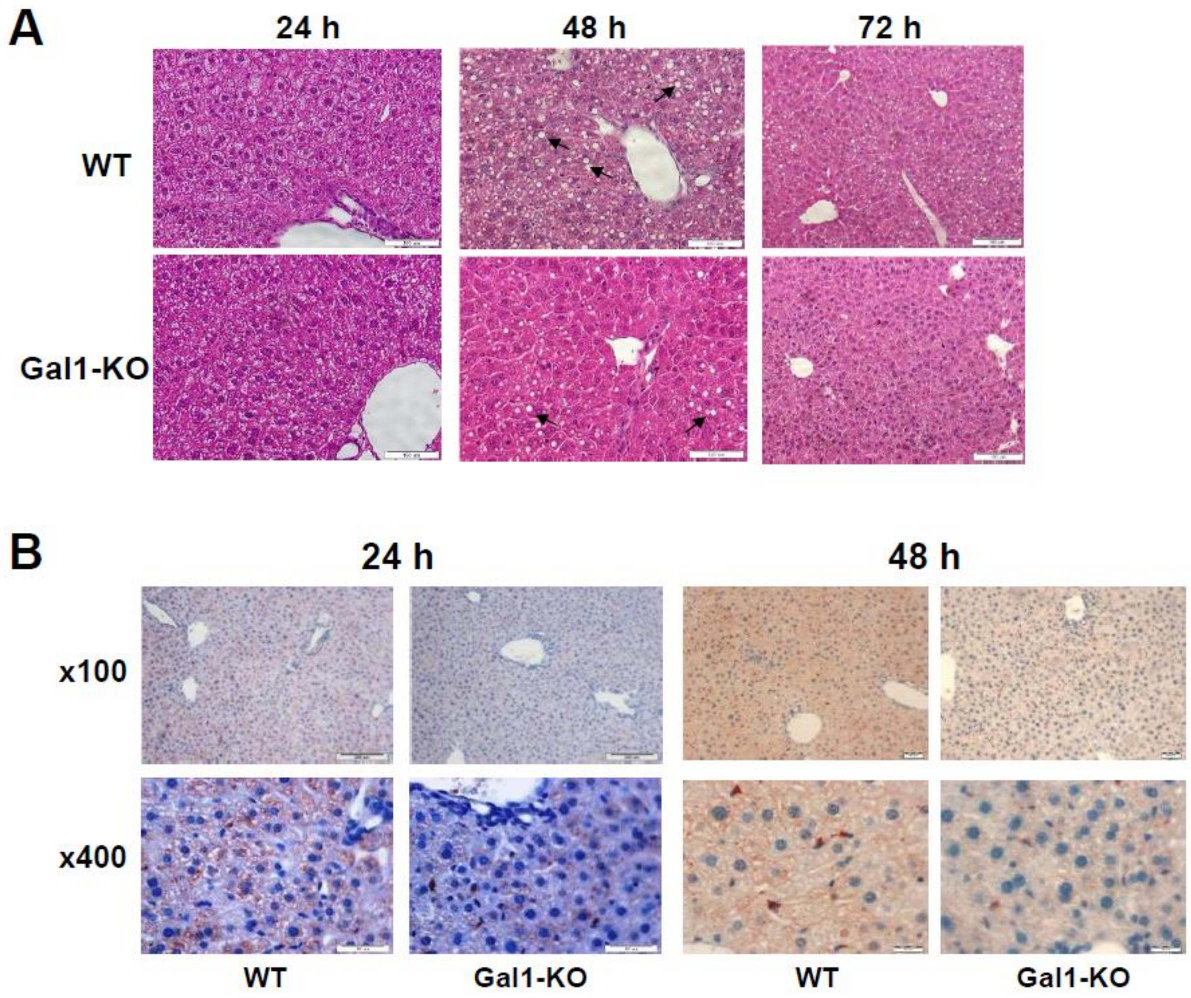

Supplementary Figure 7: Analysis of lipid accumulation in the regenerating WT and Gal1-KO livers at indicated time points following PHx. A. H&E staining of liver sections from WT (*top row*) and Gal1-KO (*bottom row*) mice taken at indicated time points post-PHx reveals a decreased accumulation of lipid droplets (indicated by arrows) in the Gal1-KO compared to WT liver. Magnification x200. **B.** Immunohistochemical staining for adipophilin of sham-operated WT and Gal1-KO mice reveals only a minor reactivity for adipophilin in the livers of both congenic strains at 24 hours and 48 hours following sham surgery. Magnification x400.

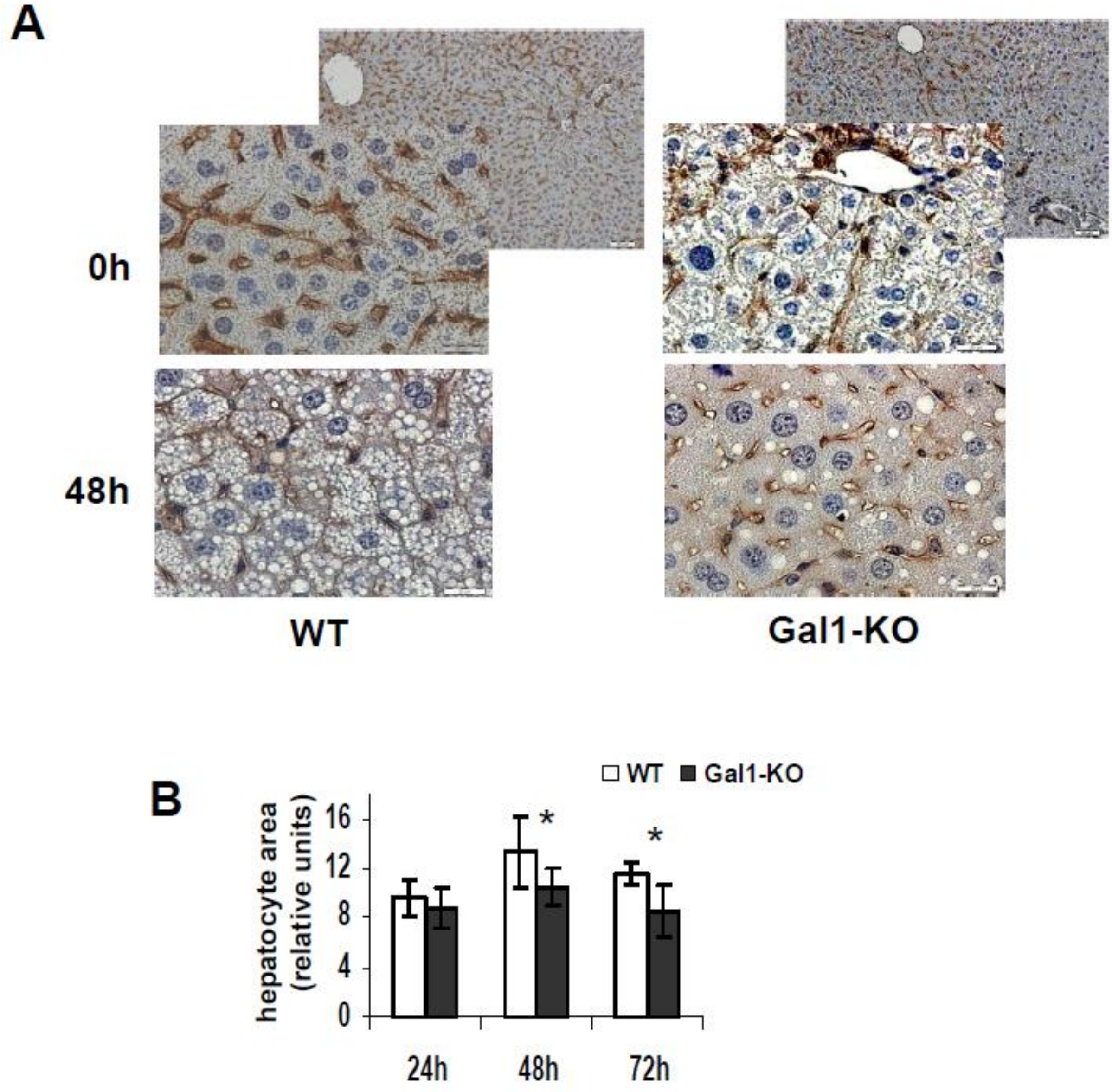

Supplementary Figure 8: Increased cell size of hepatocytes in the liver of WT versus Gal1-KO mice following PHx is associated with more pronounced adipogenesis in the WT strain. A. Immunohistochemical staining of liver tissue sections for β-catenin. Magnification: x100 (backward pictures), x400 (forward pictures). **B.** Quantification of the average size of hepatocytes (square μM) based on the images presented in (**A**). Size of hepatocytes was evaluated using Soft Imaging System CellSens Entry (Olympus, Australia) microscope equipment (in pixels, that were divided by factor 200) in the 10 HPF (4 - 5 mice per each experimental group). Cells were counted in the liver zone 2.

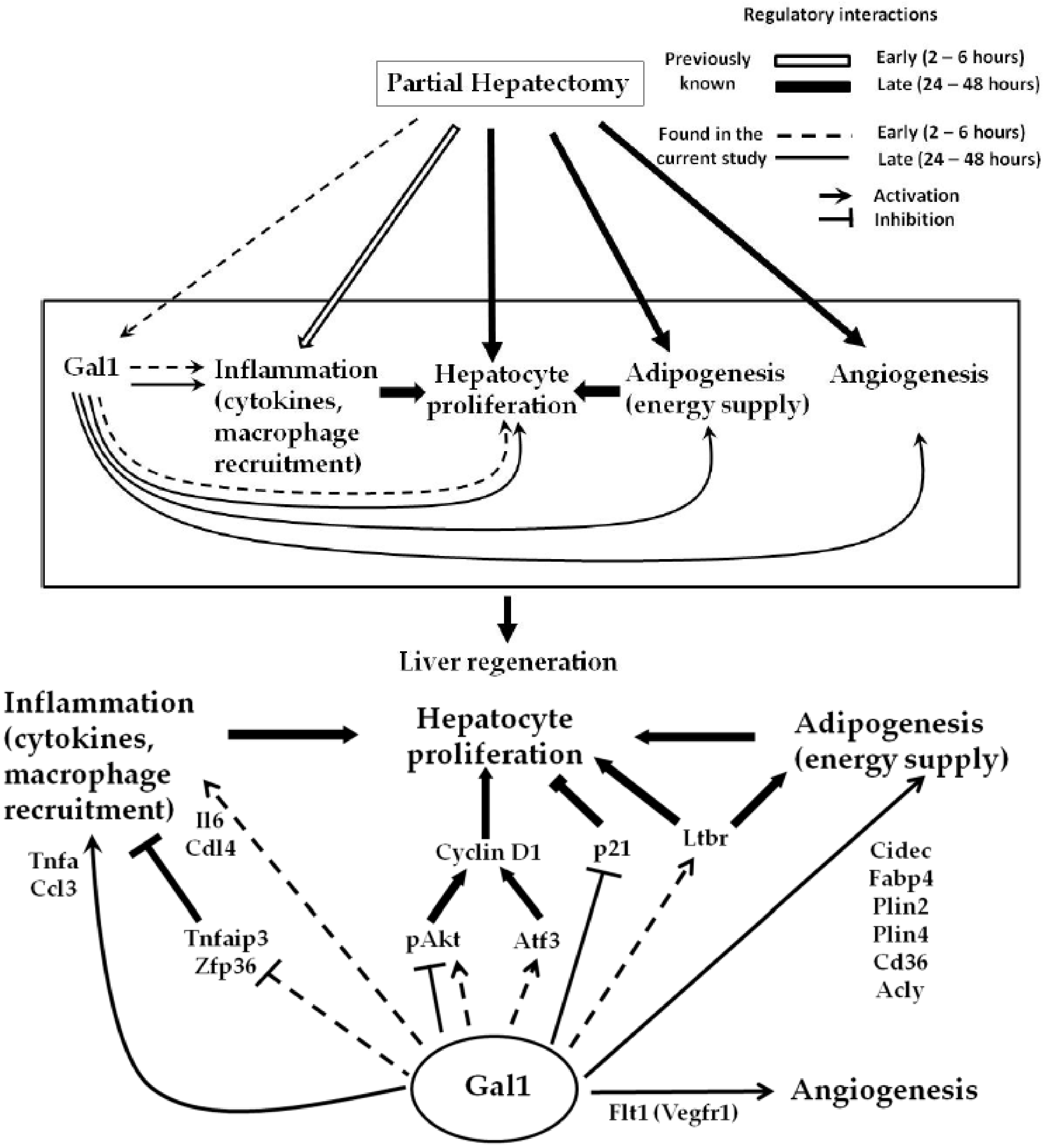

Supplementary Figure 9: Scheme of Gal1 effects on LR following PHx. Top panel – the scheme demonstrates interactions of Gal1 with different processes that comprise LR. Bottom panel – the enlarged scheme of interactions shown inside the rectangle in the top panel, demonstrating the most aberrantly expressed genes and their regulatory interactions in the Gal1-KO liver following PHx.

Supplementary Materials and Methods

Partial hepatectomy (PHx). The 70% PHx or sham surgery was performed on 11-12 weeks old non-fasting male mice. Special care has been taken to preserve the gallbladder. Each hepatectomized mouse was kept in a separate cage. The post-operation mortality was low (about 2%) and similar in both wild type and Gal1-KO mice. At least four animals from each of these two genotypes were hepatectomized for each time point.

Harvesting mouse liver tissue. At the indicated time points following laparatomy or sham surgery, mice were anesthetized with isoflurane, blood was collected from the peri-orbital sinus, and anesthetized mice were then sacrificed by cervical dislocation. When needed, mice were injected i.p. with bromodeoxyuridine (BrdU, 100mg/kg body weight, 0.2 ml; B5002, Sigma Aldrich) two hours before euthanasia. All mice were operated at the same time interval between 12:00 a.m. and 15:00 p.m. The remnant livers were rapidly excised, weighed and one part was fixed in formalin for histology; the remaining liver tissue was snap-frozen for RNA and protein analyses as described previously [1], and liver tissue was examined by a qualified pathologist (O.P.). Preoperative and regenerated liver weight was expressed as liver-to-body-weight ratio and used to calculate the relative growth of residual liver lobes as the regenerated/preoperative liver weight x100%.

Blood analysis. Serum was obtained by centrifugation of total blood at 3,200 g for 10 min. Liver damage was assessed through the ALT and ALP activities (U/L) in the serum by an enzyme assay using Reflotron (Roche, Mannheim, Germany).

Genotyping. Gal1-KO genotype was confirmed during experiments. DNA from mouse tails was purified by D-Tail^{TM} Extraction kit (Synthezza Bioscience Ltd, Jerusalem, Israel).

Genotyping was carried out by PCR analysis of DNA extracted from mouse tails as a template using primers specific to Gal1 and Neo genes [2].

Testing gene expression by semi-qRT–PCR. Reverse transcription of total liver RNA was performed using the MMLV Reverse Transcriptase and random hexamer primers (Promega, Madison, WI). Semi-quantitative PCR was performed using 2xKapa2GFast Taq Polymerase (KM5101, Kappa Biosciences, Norway). For each gene, the cDNA concentration and the number of PCR cycles were established in the linear amplification range. Expression levels for each gene were normalized against housekeeping genes Mrps21 or Hprt. The primers were designed using the NCBI Primer-BLAST tool (see Supplementary Table 2) and were produced by Syntezza Bioscience Ltd. (Jerusalem, Israel). Pictures of agarose gels were taken by B.I.S. 202D BioImaging System (Amersham Pharmacia Biotech., USA). The intensities of bands were quantified using the Scion Image program (Scion Corp., Frederick MD, NIH).

Testing gene expression by qRT–PCR. Reverse transcription of total RNA was performed using the qScript™ cDNA Synthesis Kit (#95047) and Perfecta Sybr Green Fast Mix ROX (#95073) (both Quanta BioSciences Inc., Gaithersburg, MD, USA). qPCR assay was performed on an AB 7900 HT fast real-time PCR system (Applied Biosystems, Foster City, CA, USA) or CFX384 ™ Real-Time System with C1000 Touch Thermal Cycle (BioRad, Hercules, CA, USA).

References

[1] Potikha T, Stoyanov E, Pappo O, Frolov A, Mizrahi L, Olam D, et al. Interstrain differences in chronic hepatitis and tumor development in a murine model of inflammation-mediated hepatocarcinogenesis. Hepatology 2013;58:192-204.

[2] Poirier F, Robertson EJ. Normal development of mice carrying a null mutation in the gene encoding the L14 S-type lectin. Development 1993;119:1229-1236.
